# Atlanta Medical College Commencement

**Published:** 1890-04

**Authors:** 


### ATLANTA MEDICAL COLLEGE COMMENCEMENT.

The thirty-second annual commencement of the Atlanta Medical College, which took place last night at DeGive's opera house, attracted a great crowd.

Rev. Dr. John W. Heidt opened the exercises by prayer, during which the audience remained standing.

### THE PROCTOR'S REPORT.

Dr. W. S. Kendrick, the proctor of the college, then read the following report:

ATLANTA, Ga., March 6, 1890.—To the Board of Directors of the Atlanta Medical College—Gentlemen: the session of 1889-

'90 has been an exceedingly prosperous one for the institution—the class being the largest in the history of the school, and the revenues greater than ever before. New features have been introduced, and no pains spared to bring the teaching up to the standard of the best medical schools in the country.

The number of matriculants reached 135—sixty-five seniors and seventy juniors. Of these Georgia furnished 100, Alabama 18, South Carolina 5, Texas 4, North Carolina 3, Arkansas 1, Florida 1, Wisconsin 1, Mississippi 1 and Mexico 1.

The following gentlemen—fifty-one in number—have passed satisfactory examinations and ask for the degrees of "Doctor of Medicine." They are sober, industrious and moral, and we hope will maintain, with dignity and honor, the profession at whose door they now knock for admittance.

THE GRADUATING CLASS.

E. M. Baily, G. B. Ballenger, M. J. Banks, J. C. Bennett, H, M.; D. B. Bosworth, J. T. Boykin, E. B. Bush, A. T. Calhoun, W. R. Camp, M. S. Chandler, H. W. Clements, G. A. Davis, A. J. Farley, J. E. Flowers, W. B. Goodman, R. J. Goodman, L. C. Furr, A. S. Garrett, J. A. Gibson, W. A. Goode, W. R. George, J. C. Guffies, J. F. Hall, H. T. Hodges, J. R. G. Howell, G. B. Hyde, W. H. Inghram, W. Mathews, W. F. Love, R. C. Loyd, W. P. Magruder, A. J. Matthews, J: H. McGuffey, W. A. Monnish. R N Pitts, J P Presscott, J A Quillian, A B Robinson. T B Selman, R S Spears, H M H C Strickland, J C Swann, W R Terry, A S Tucker, J R Tucker, A J Tuggle, W C Warren, W J Wheeler, E F Woods, L K Burruss, M D, adeundem, J H Reid, deceased.

Judge Marshall Clarke delivered the documents, and when each of the fifty-one gentlemen had received the prize for which he had labored for two years, he made a telling speech congratulating them on the result of their work, and urging them not to abate their efforts until they had achieved the success in life which was attainable by all hard-working, honest, professional men.

Mr. Hooper Alexander delivered the annual oration, and his eloquent words evidently had great effect on his hearers.

Dr. J. A. Quillian delivered the valedictory address. After speaking of the necessities of life, Dr. Quillian then reviewed the past of his class and anticipated the unknown future which was opening out to his classmates.

In conclusion Dr. Quillian appealed to his classmates to lead such lives that when the end came, as come it would, they would be received in future life with the words, "Well done, thou good and faithful servant."

Colonel T. P. Westmoreland delivered the prizes to the three honor men. Dr. D. B. Bosworth received the first honor, Dr. W. P. McGruder the second, Dr. E. M. Baily the third honor. Dr. Kendrick stated, however, that he wished to make honorable mention of Doctors J. C. Bennett and R. S. Speers, as only a quarter of a vote had prevented their sharing in an honor.

Rev. James W. Pogue delivered the benediction, after which the audience dispersed amidst general expressions of pleasure at the success of the exercises.

